# Safety, tolerability, pharmacokinetics, pharmacodynamics, and efficacy of WBP216, a novel IL-6 monoclonal antibody, in patients with rheumatoid arthritis: A phase Ia randomized placebo-controlled study

**DOI:** 10.3389/fimmu.2022.1110992

**Published:** 2023-02-28

**Authors:** Xiaomei Leng, Xiange Tang, Pei Hu, Xiaoduo Guan, Qian Li, Cipo Huang, Qiang Zhang, Rui Chen, Xiaofeng Zeng

**Affiliations:** ^1^ Department of Rheumatology, Peking Union Medical College Hospital, Chinese Academy of Medical Science & Peking Union Medical College, Beijing, China; ^2^ Clinical Pharmacology Research Center, Peking Union Medical College Hospital, State Key Laboratory of Complex Severe and Rare Diseases, NMPA Key Laboratory for Clinical Research and Evaluation of Drug, Beijing Key Laboratory of Clinical PK & PD Investigation for Innovative Drugs, Chinese Academy of Medical Sciences & Peking Union Medical College, Beijing, China; ^3^ WuXi Clinical Development Services Co., Ltd, Wuxi, China

**Keywords:** monoclonal antibody, rheumatoid arthritis, WBP216, safety, placebo-controlled

## Abstract

**Background:**

WBP216 is a novel human immunoglobulin G1 (IgG1) monoclonal antibody for interleukin (IL)-6. We aimed to assess the safety, tolerability, pharmacokinetics (PK), and pharmacodynamics (PD) of a single ascending dose (SAD) of WBP216 in patients with rheumatoid arthritis (RA).

**Methods:**

In this double-blind, placebo-controlled, SAD, phase Ia study, patients with RA were randomized in a 3:1 (Group A1, 10 mg) and 6:2 (Group A2, 30 mg; Group A3, 75 mg; Group A4, 150 mg; Group A5, 300 mg) ratios to receive either ascending doses of WBP216 or placebo subcutaneously. The primary endpoint was the incidence of adverse events (AEs), while the secondary endpoints were characterization of PK, PD, and immunogenicity of WBP216 and the exploratory endpoints included improvements in RA clinical metrics. All statistical analyses were performed using SAS^®^ version 9.2.

**Results:**

A total of 41 subjects (34 females and 7 males) were enrolled in the study. WBP216 was well tolerated in all doses (10-300 mg). Most treatment-emergent AEs (TEAEs; 97.6%) were of grade 1 severity and resolved without any treatment. No subjects experienced TEAEs leading to withdrawal or death during the study. An increase in serum concentration and total IL-6 from baseline was observed, while a substantial decrease in high-sensitivity C-reactive protein (hs-CRP) and erythrocyte sedimentation rate (ESR) was observed in all the WBP216 groups. Anti-drug antibodies were detected in only one subject after dosing, indicating an acceptable immunogenicity profile. Limited ACR20 and ACR50 response was observed in the WBP216 groups and no response in the placebo group.

**Conclusion:**

WBP216 demonstrated a good safety profile and evidence of potential efficacy in the treatment of patients with RA.

**Clinical trial registration:**

http://www.chinadrugtrials.org.cn/clinicaltrials.searchlistdetail.dhtml, identifier CTR20170306.

## Introduction

Rheumatoid arthritis (RA) is a chronic, systemic inflammatory, autoimmune disease characterized by synovitis, inflammation, progressive joint damage, and deformity with a prevalence ranging from 0.4% to 1.3% (1). The therapeutic management paradigm for RA has been augmented by the introduction of targeted biologics that focus on immune and inflammatory processes (2, 3). Despite marked improvements with biologics in patients with RA, only a minority of patients achieved adequate disease control (4). Furthermore, they are associated with serious adverse events (SAEs) and an increase in the risk of serious infections (5, 6). Thus, there remains an unmet need for the treatment of patients with RA and warrants the development of new targeted drugs.

Interleukin (IL)-6 is a multipotent cytokine that plays an important role in immune function, inflammatory function, hematopoiesis, and tumor formation (7). Aberrant IL-6 expression and dysregulation are typical features and important etiological factors of RA (8). Studies have confirmed that IL-6 can trigger chondrocytes and synoviocytes to produce matrix metalloproteinase (MMP)-1, MMP-3, and MMP-13, causing damage to the cartilage (9, 10). Therefore, the inhibition of IL-6 signaling pathway will be helpful in reducing inflammation and pain in patients with RA (11). Furthermore, the European League Against Rheumatism (EULAR) guidelines have indicated that IL-6 inhibitors may offer some advantages over other biologics if patient is intolerable or contraindicated to conventional synthetic disease modifying anti-rheumatic drugs (csDMARDs) (12). Tocilizumab and sarilumab are anti-IL-6 receptor antibodies that bind to IL-6 receptors (13) and reduce disease activity with clinical significance, ultimately inhibiting the process of joint damage (14–16). However, some of its side effects such as infections, neutropenia, increase in serum cholesterol, transient decrease in neutrophil count, and abnormal liver function test results limit the clinical application of these drugs (17, 18).

WBP216 is a novel human immunoglobulin G1 (IgG1)-YTE (IgG1 triple mutation) monoclonal antibody for IL-6. The strong affinity of WBP216 prevents the interaction between IL-6 and its receptor, thereby reducing the proinflammatory activities. In addition WBP216 can directly inhibit the production of C-reactive protein (CRP) and erythrocyte sedimentation rate (ESR) resulting in an improvement in the swollen and tender joints in patients with RA (19). Preclinical studies demonstrated a higher affinity of WBP216 for IL-6 receptors and a longer half-life compared to other IL-6 inhibitors. The longer half-life of WBP216 is due to YTE (M252Y/S254T/T256E) mutations in the fragment crystallization (Fc) region (20), which facilitates subcutaneous administration, thereby reducing the frequency of administration and improving the patient compliance to treatment. Based on these observations, we intended to assess the safety, tolerability, pharmacokinetics (PK), and pharmacodynamics (PD) of single ascending dose (SAD) of subcutaneously administered WBP216 in Chinese patients with RA. This study investigated whether the extended half-life observed in animal studies can be maintained in humans, and whether the clinical efficacy can be obtained in humans by subcutaneous dose of WBP216.

## Methods

### Study design

This was a randomized (within-group), double-blind, placebo-controlled, SAD, phase Ia study including patients with RA from Peking Union Medical College Hospital, Chinese Academy of Medical Sciences and Beijing Hospital. The trial was registered in the Centre for Drug Evaluation of China Food and Drug Administration (CFDA) (ChiCTR: CTR20170306). The trial was conducted in compliance with the Declaration of Helsinki, as well as with Good Clinical Practice and applicable regulatory requirements. The protocol was approved by the ethic committee of Peking Union Medical College Hospital, Chinese Academy of Medical Sciences and Beijing Hospital (site 1 approval number 2017BJYYEC-019-02/site 2 approval number: HS2017014/NMPA approval number: 2016L10654) before study initiation and written informed consent was obtained by a delegated rheumatologist from all study patients before performing any study procedure.

### Study population

Eligible patients were males or females diagnosed with RA (according to the 2010 American College of Rheumatology [ACR]/EULAR criteria for at least 6 months and on anti-RA treatments for 12 weeks, without significant concomitant illness, recent severe infections or organ dysfunction) (21) and aged 18 to 70 years, with a body mass index (BMI) of 19.0 to 30.0 kg/m^2^. Only the patients with active RA (subjects who had ≥ 2 swollen joints in 66 joints, and ≥ 4 tender joints in 68 joints) at screening and baseline were included. Patients on oral prednisolone (≤10 mg/day), methotrexate (7.5-25 mg/week), hydroxychloroquine (200-400 mg/day), leflunomide (10-20 mg/day), and sulfasalazine (2-3 g/day) were considered if they were on stable dose for at least 4 weeks before screening. Patients currently taking non-steroidal anti-inflammatory drugs were required to be on a stable dose for at least 2 weeks prior to screening.

Key exclusion criteria were history of and/or current clinically significant illness that had not been stable for 3 months prior to enrollment, or an acute illness, planned medical/surgical procedure, or trauma within 2 months prior to enrollment, use of traditional Chinese medicines, over-the-counter emergency anti-inflammatory drugs, or any active/attenuated vaccine within 4 weeks prior to screening, current or previous use of IL-6 antagonists, or other biological modifying anti-rheumatics within 12 weeks or more (as required) prior screening, presence of other systemic inflammatory conditions (eg, systemic lupus erythematosus, spondyloarthropathy, systemic vasculitis, gout, and systemic vasculitis), any acute, chronic, or recurrent infections (eg, recurrent sinusitis, genital herpes, herpes zoster, osteomyelitis, and urinary tract infections) during the screening period, subjects who were positive for human immunodeficiency virus, hepatitis C virus antibody, or hepatitis B surface antigen and those participating in other clinical trials.

### Treatment

**Figure 1:**
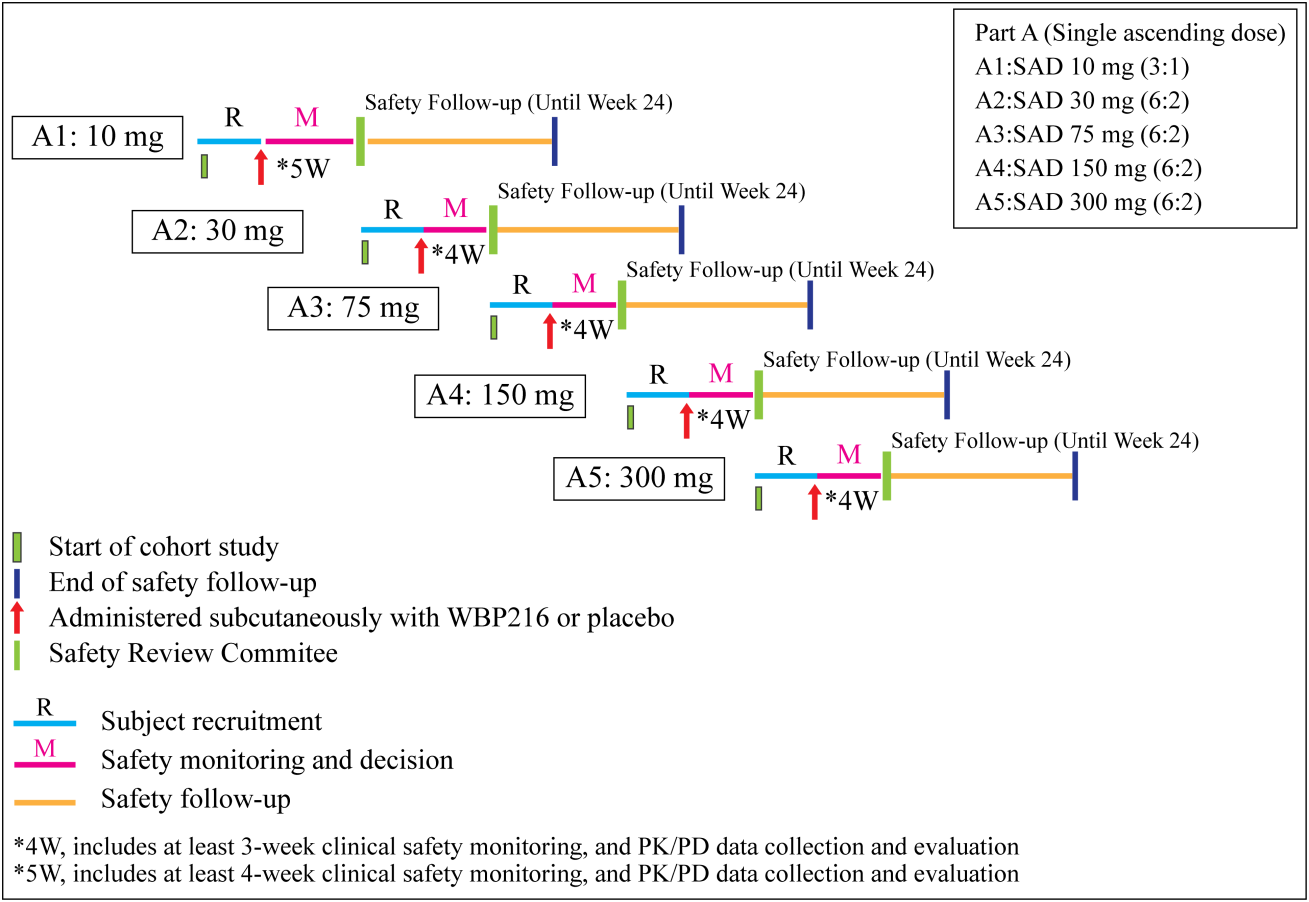
Schematic presentation of the study. W, week; SAD, single ascending dose; PK, pharmacokinetic; PD, pharmacodynamics.

The study included a screening period (week 4 to week 1), a safety monitoring period (4 weeks for Group A1, and 3 weeks for Groups A2-A5), and a safety follow-up period (up to week 24) (**Figure 1**). The randomization sequence and allocation were accomplished using sealed envelopes containing a computer-generated sequence. The eligible subjects were divided in to 6 groups: Group A1, 10 mg of WBP216; Group A2, 30 mg of WBP216; Group A3, 75 mg of WBP216; Group A4, 150 mg of WBP216; Group A5, 300 mg of WBP216; and matching placebo. On Day 1 of study, eligible subjects were randomized in 3:1 ratio (Group A1) and in 6:2 ratio (Group A2 to A5) to receive either ascending doses of WBP216 or placebo. The WBP216 doses were administered subcutaneously to the patients in the morning.

After review (including but not limited to reported adverse events [AEs], vital signs, electrocardiogram [ECG], and clinical laboratory test results) and evaluation of safety data with reference to the available PK/PD data by the Independent Safety Review Committee (iSRC), it was decided to conduct a further dose escalation and safety monitoring period. All study personnel remained blinded to treatment until study completion.

### Outcomes and endpoints

The outcomes assessed in the study were safety, tolerability, PK, PD, and efficacy of WBP216. The primary endpoint was the incidence of AEs associated with escalating doses of WBP216 while the secondary endpoints were the assessment of PK, PD, and the immunogenicity of WBP216. The exploratory endpoint was effectiveness of WBP216 in treating RA.

#### Safety assessments

Safety and tolerability were assessed in terms of AEs and serious AEs, physical examinations, vital signs, using ECG, and clinical laboratory tests (including blood biochemistry, hematology, urinalysis, serology, pregnancy tests, and confirmation of menopausal status tests) and tuberculosis (TB) tests.

#### PD, PK, and immunogenicity assessments

PD assessment was performed by measuring total IL-6, free IL-6, high-sensitivity CRP (hs-CRP), and ESR. The PK assessment was conducted by non-compartmental analysis using WinNonlin (version 6.3; Pharsight Corp., Mountain View, CA, USA) and the parameters such as maximum serum concentration (C_max_), time to reach C_max_ (T_max_), terminal half-life (t_1/2_), area under the concentration-time curve from time 0 to t (AUC_0-t_), area under the concentration-time curve from time 0 to infinity (AUC_0-∞_), systemic clearance (CL/F) and apparent volume of distribution (V_d_/F) were measured. An immunogenicity ELISA was developed to quantify the antibody response to WBP216 in human serum. The potential immunogenicity of WBP216 was assessed by summarizing the number and percentage of subjects who were positive for anti-drug antibody (ADA) by dose groups. PD, PK, and immunogenicity were assessed on day 1 (pre-dose), 2, 4, 8, 15, 22, 29, 43, 57, 85, 113, 141, and 169.

#### Efficacy assessments

Efficacy was analyzed based on American College of Rheumatology 20% (ACR20) and 50% (ACR50) response criteria (22). ACR20 is defined as a 20% improvement in the number of tender and swollen joints (out of 68 joints and 66 joints, respectively) and 20% improvement in 3 of the following 5 criteria: patient’s global assessment of disease activity on a 0 to 100 visual analogue scale (VAS), physician’s global assessment of disease activity on a 0 to 100 VAS, patient’s assessment of pain on a 0 to 100 VAS (1-week review period), subject’s assessment of physical function (health assessment questionnaire-disability index [HAQ-DI]), acute phase reactants (ESR, hs-CRP) while ACR50 is defined as 50% improvement in all the above parameters. The efficacy was also measured in terms of Disease Activity Score 28-joint assessment (DAS28) (23).

### Statistical analysis

Given the exploratory nature of this study, no calculation was performed for sample size estimation. The number of subjects was determined according to the requirement of the China Food and Drug Administration (CFDA) for PK studies. The safety analysis set was defined as all patients who received at least one dose of the investigational treatment. The intent-to-treat analysis set was identified as all patients who were randomized. The per protocol analysis set included patients who received all study administrations and had complete and valid data in all study evaluations.

Demographics, safety, PK, PD, and biomarker data were summarized descriptively by dose group. Continuous efficacy parameters were summarized by timepoint, using changes from baseline, while the categorical parameters were summarized using only frequencies and incidences. Mean and individual concentration-time profiles were presented graphically as appropriate. The occurrence of ADAs was presented as frequency and percentage of subjects. ACR20 and ACR50 response rates and changes in DAS28 score from baseline were presented by the dose group using frequencies, percentages, and 95% confidence intervals (CIs). The AEs were described with preferred terms and classified into system organ classifications (SOCs) based on MedDRA version 19.0. All statistical analyses were performed using SAS^®^ version 9.2 (SAS Institute, Cary, North Carolina, USA).

## Results

### Patient disposition and baseline characteristics

A total of 143 subjects were screened, of whom 102 subjects failed screening and 41 subjects were included in the study. All subjects enrolled were of Han race, consisting of 7 (17.1%) male and 34 (82.9%) female subjects with a mean age (standard deviation [SD]) of 49.5 (9.50) years (range: 26 to 68 years). The subjects had a mean weight (SD) and mean BMI (SD) of 61.32 (8.758) kg (range: 47.0 to 77.6 kg), and 23.66 (2.745) kg/m^2^ (range: 19.1 to 29.7 kg/m^2^) respectively (**Table 1**). A total of 3, 10, 6, 6, 6, and 10 subjects were randomized to receive WBP216 10 mg, 30 mg, 75 mg, 150 mg, 300 mg, and placebo, respectively. All the 41 subjects enrolled in the study were included in the full analysis set (FAS) and safety analysis set (SS). Due to major protocol deviation, 4 subjects included in WBP216 30 mg group and 1 subject in placebo group were excluded from the PK analysis set (PKAS), PD analysis set (PDAS), immunogenicity analysis set (ADAS), and efficacy analysis set (EAS), while subjects receiving placebo were not included in the PKAS, hence, a total of 36 (87.8%) subjects were included in the PDAS, ADAS, and EAS and 27 (65.9%) subjects were included in the PKAS. At baseline, no subjects in the WBP216 and placebo groups had DAS28 scores ≤ 3.2, 13 (36.1%) subjects had DAS28 scores > 3.2 and ≤ 5.1, and 23 (63.9%) subjects had DAS28 scores > 5.1. Past medical and medications history along with concomitant medications of enrolled subjects are presented in **Supplementary Table 1**.

**Table 1:** Demographics and clinical characteristics: Full analysis set.

Demographics and Characteristics	10 mg WBP (N =3) n (%)	30 mg WBP (N =10) n (%)	75 mg WBP (N =6) n (%)	150 mg WBP (N =6) n (%)	300 mg WBP (N =6) n (%)	Placebo (N =10) n (%)	Total (N =41) n (%)
Age (Years)							
Mean (SD)	46.7 (6.11)	48.2 (12.19)	46.7 (8.96)	54.0 (5.90)	50.2 (13.23)	50.1 (7.67)	49.5 (9.50)
Median (Q1, Q3)	48.0 (40.0, 52.0)	52.0 (42.0, 56.0)	46.0 (45.0, 52.0)	51.5 (51.0, 58.0)	48.5 (38.0, 61.0)	53.0 (46.0, 55.0)	52.0 (44.0, 56.0)
Minimum, Maximum	40, 52	26, 64	32, 59	48, 64	37, 68	38, 61	26, 68
Missing (n)	0	0	0	0	0	0	0
Gender [n (%)]							
Male	1 (33.3)	3 (30.0)	1 (16.7)	1 (16.7)	0	1 (10.0)	7 (17.1)
Female	2 (66.7)	7 (70.0)	5 (83.3)	5 (83.3)	6 (100)	9 (90.0)	34 (82.9)
Missing	0	0	0	0	0	0	0
Ethnic [n (%)]							
Han	3 (100)	10 (100)	6 (100)	6 (100)	6 (100)	10 (100)	41 (100)
Other	0	0	0	0	0	0	0
Missing	0	0	0	0	0	0	0
Height (cm)							
Mean (SD)	163.0 (4.58)	160.1 (6.37)	160.0 (10.5)	162.8 (6.1)	161.3 (5.8)	159.9 (6.9)	160.8 (6.7)
Median (Q1, Q3)	162.0 (159.0, 168.0)	159.0 (157.0, 160.0)	156.0 (153.0, 163.0)	161.0 (160.0, 162.0)	159.5 (158.0, 161.0)	158.0 (155.0, 165.0)	160.0 (157.0, 162.0)
Minimum, Maximum	159, 168	151, 171	152, 180	158, 175	157, 173	153, 175	151, 180
Missing	0	0	0	0	0	0	0
Weight(kg)							
Mean (SD)	58.57 (4.0)	64.80 (8.7)	63.33 (11.9)	63.30 (8.0)	61.68 (9.3)	56.06 (6.7)	61.32 (8.7)
Median (Q1, Q3)	57.10 (55.50, 63.10)	65.50 (58.00, 71.00)	61.15 (52.60, 76.70)	59.35 (57.80, 73.00)	62.75 (56.00, 68.00)	53.85 (52.00, 60.10)	58.50 (55.00, 68.00)
Minimum, Maximum	55.5, 63.1	52.0, 76.0	50.8, 77.6	56.1, 74.2	47.0, 73.6	47.3, 71.0	47.0, 77.6
Missing	0	0	0	0	0	0	0
BMI (kg/m^2^)							
Mean (SD)	22.03 (0.814)	25.25 (2.906)	24.60 (2.712)	23.80 (2.123)	23.67 (3.199)	21.90 (2.213)	23.66 (2.745)
Median (Q1, Q3)	22.40 (21.10, 22.60)	25.05 (23.20, 27.70)	23.70 (22.80, 26.80)	23.30 (22.30, 24.20)	23.55 (22.40, 24.60)	21.45 (20.80, 22.30)	22.80 (21.80, 24.60)
Minimum, Maximum	21.1, 22.6	20.3, 29.7	21.7, 28.9	21.9, 27.8	19.1, 28.8	19.9, 27.7	19.1, 29.7
Missing	0	0	0	0	0	0	0

BMI, body mass index; Q1, 1st quartile; Q3, 3rd quartile; SD, standard deviation.

### Safety

Overall, a total of 266 AEs were reported, in 40 (97.6%) subjects (except 1 subject from placebo group), of which 254 were treatment-emergent AEs (TEAEs). A total of 168 drug-related AEs were reported in 34 (82.9%) subjects with 3 (100%), 6 (60.0%), 6 (100%), 5 (83.3%), 5 (83.3%), and 9 (90.0%) subjects from WBP216 10|, 30, 75, 150, 300 mg, and placebo groups, respectively. One subject (A205) from WBP216 30 mg group experienced serious AE (SAE) (myocardial bridging) of Grade 1 severity, which was resolved with no concomitant medication/non-pharmacological therapy indicating SAE non-relevant to the study drug.

Most TEAEs (40 subjects, 97.6%) reported in the study were of grade 1 severity. Grade 2 TEAEs were reported by 20 (48.8%) patients, whereas only 2 (4.9%) subjects experienced Grade 3 TEAEs (upper respiratory tract infection, and decrease in neutrophil count). No subjects reported Grade 4 TEAEs and TEAEs that led to withdrawal from the study, or deaths during the study. The TEAEs with total incidence of ≥5.0% (WBP216 vs placebo) are presented in **Table 2**.

**Table 2:** Treatment-emergent adverse events (with total incidence ≥5.0%): Safety analysis set.

Preferred Term	10 mg WBP (N = 3)			30 mg WBP (N = 10)			75 mg WBP (N = 6)			150 mg WBP (N = 6)			300mg WBP (N=6)			WBP216 (N = 31)			Placebo (N = 10)			Total (N = 41)		
	n1	n	(%)	n1	n	(%)	n1	n	(%)	n1	n	(%)	n1	n	(%)	n1	n	(%)	n1	n	(%)	n1	n	(%)
Upper respiratory tract infection	2	2	66.7	1	1	10.0	2	2	33.3	2	1	16.7	5	4	66.7	12	10	32.3	4	4	40.0	16	14	34.1
Aspartate aminotransferase increased	2	2	66.7	0	0	0	1	1	16.7	5	4	66.7	3	3	50.0	11	10	32.3	2	2	20.0	13	12	29.3
Neutrophil count decreased	2	2	66.7	1	1	10.0	3	3	50.0	3	3	50.0	2	2	33.3	11	11	35.5	1	1	10.0	12	12	29.3
Alanine aminotransferase increased	2	2	66.7	1	1	10.0	0	0	0	3	2	33.3	3	2	33.3	9	7	22.6	1	1	10.0	10	8	19.5
Blood alkaline phosphatase increased	1	1	33.3	0	0	0	3	3	50.0	0	0	0	1	1	16.7	5	5	16.1	3	2	20.0	8	7	17.1
Leukopenia	0	0	0	4	3	30.0	0	0	0	0	0	0	3	1	16.7	7	4	12.9	2	2	20.0	9	6	14.6
Blood creatine phosphokinase increased	0	0	0	5	4	40.0	0	0	0	0	0	0	0	0	0	5	4	12.9	1	1	10.0	6	5	12.2
Blood thyroid stimulating hormone increased	1	1	33.3	0	0	0	1	1	16.7	1	1	16.7	1	1	16.7	4	4	12.9	1	1	10.0	5	5	12.2
Haemoglobin decreased	1	1	33.3	0	0	0	2	2	33.3	0	0	0	0	0	0	3	3	9.7	2	2	20.0	5	5	12.2
Reticulocyte percentage increased	0	0	0	3	3	30.0	0	0	0	0	0	0	2	1	16.7	5	4	12.9	1	1	10.0	6	5	12.2
White blood cell count decreased	0	0	0	0	0	0	3	2	33.3	2	2	33.3	1	1	16.7	6	5	16.1	0	0	0	6	5	12.2
Dizziness	0	0	0	0	0	0	2	2	33.3	2	2	33.3	1	1	16.7	5	5	16.1	0	0	0	5	5	12.2
Blood bilirubin increased	0	0	0	0	0	0	1	1	16.7	1	1	16.7	3	2	33.3	5	4	12.9	0	0	0	5	4	9.8
Oropharyngeal pain	0	0	0	0	0	0	0	0	0	1	1	16.7	1	1	16.7	2	2	6.5	2	2	20.0	4	4	9.8
Blood triglycerides increased	0	0	0	0	0	0	1	1	16.7	1	1	16.7	1	1	16.7	3	3	9.7	0	0	0	3	3	7.3
Gamma-glutamyltransferase increased	0	0	0	0	0	0	1	1	16.7	2	2	33.3	0	0	0	3	3	9.7	0	0	0	3	3	7.3
Low density lipoprotein increased	0	0	0	0	0	0	0	0	0	3	2	33.3	1	1	16.7	4	3	9.7	0	0	0	4	3	7.3
Hypercholesterolaemia	0	0	0	2	1	10.0	0	0	0	1	1	16.7	1	1	16.7	4	3	9.7	0	0	0	4	3	7.3
Thyroid disorder	0	0	0	3	3	30.0	0	0	0	0	0	0	0	0	0	3	3	9.7	0	0	0	3	3	7.3

n1 = number of events; n = number of subjects.

MedDRA version 19.0 was used to code adverse events.

### Pharmacodynamics

**Figure 2:**
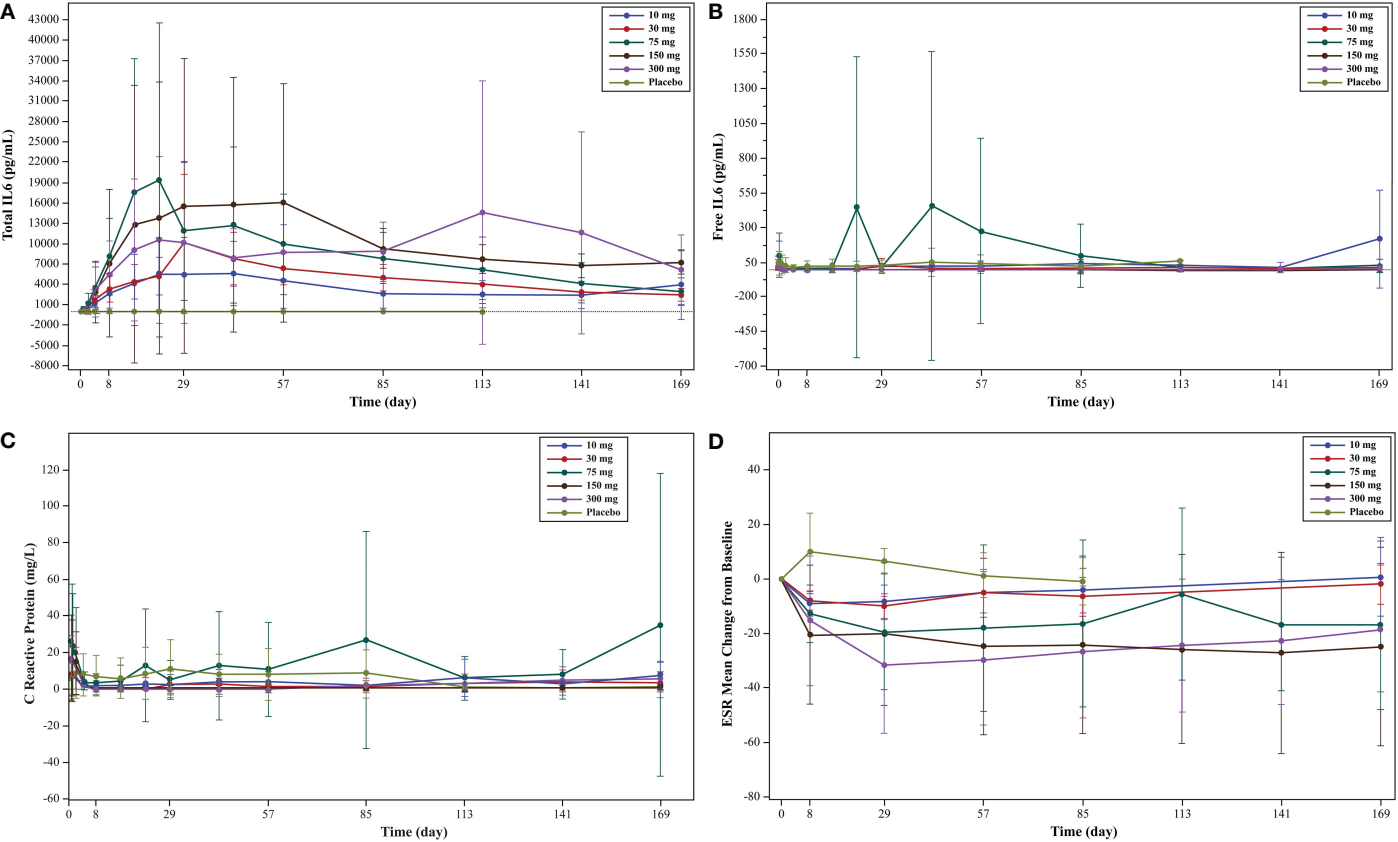
**(A)** Mean serum concentration of total IL-6 (linear scale) levels: pharmacodynamic analysis set; **(B)** mean serum concentration of free IL-6 (linear scale) levels pharmacodynamic analysis set; **(C)** mean serum concentration of hs-CRP (linear scale) levels; **(D)** ESR mean change from baseline in each cohort: efficacy analysis set. IL, interleukin; ESR, erythrocyte sedimentation rate.

An increase in total IL-6 levels from baseline was observed in all WBP216 groups (**Figure 2A**). Though free IL-6 levels decreased after WBP216 dosing, the levels returned to normal (baseline) in approximately 1 to 2 weeks (**Figure 2B**). The mean hs-CRP substantially decreased with WBP216 treatment and was maintained at low-level throughout, except for 75 mg group due to an extremely high post-treatment hs-CRP level of one subject in this group. No decrease of hs-CRP was observed in the placebo group (**Figure 2C**). ESR decreased in all WBP216 groups, and a preliminary dose-response trend was observed (**Figure 2D**).

### Pharmacokinetics

**Figure 3:**
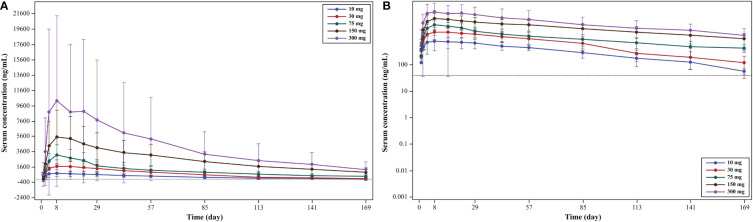
**(A)** Mean serum concentration (linear scale): pharmacokinetic analysis set; **(B)** mean serum concentration (log-linear scale): pharmacokinetic analysis set.

From mean serum concentration-time curves, the serum concentration of WBP216 was found to increase with the increase in dose in treatment groups (10-, 30-, 75-, 150- and 300-mg dose groups; **Figures 3A, B**). With increase in the WBP216 dose, AUC_0-∞_, AUC_0-t_, and C_max_ of serum WBP216 levels were found to increase. The t_1/2_ in treatment groups ranged from 980.39 to 1491.67 h. A slight increase in CL/F and V_d_/F was observed with increase in the WBP216 dose (**Table 3**).

**Table 3:** Summary statistics for serum pharmacokinetic parameters: Pharmacokinetic analysis set.

PK Parameters	Arithmetic Mean ± SD (CV%)				
	10 mg WBP (N = 3)	30 mg WBP (N = 6)	75 mg WBP (N = 6)	150 mg WBP (N = 6)	300 mg WBP (N = 6)
AUC_0-∞_ (h*µg/mL)	1451.9831 ± 585.0388 (40.3)	3066.4636 ± 1336.3662 (43.6)	4881.3872 ± 2545.6303 (52.1)	12580.4475 ± 6375.8604 (50.7)	18813.4623 ± 18534.7447 (98.5)
AUC_0-t_ (h*µg/mL)	1370.7466 ± 565.2922 (41.2)	2891.3814 ± 1188.2860 (41.1)	4238.8420 ± 2119.6180 (50.0)	10351.3446 ± 5101.3888 (49.3)	17017.0096 ± 17022.2606 (100.0)
C_max_ (ng/mL)	819.5940 ± 435.3851 (53.1)	1797.8695 ± 753.6329 (41.9)	3265.3065 ± 1254.5347 (38.4)	5648.3673 ± 3412.7529 (60.4)	10616.5207 ± 10969.5441 (103.3)
T_max_ (h)*	167.880 (167.65, 336.00)	333.985 (167.40, 670.68)	168.000 (168.00, 336.00)	240.035 (168.00, 504.00)	168.000 (72.20, 504.00)
t_1/2_ (h)	980.3914 ± 68.4887	907.9566 ± 160.2069	1106.1574 ± 559.2789	1491.6678 ± 435.7343	1077.2854 ± 288.6073
CL/F (mL/h)	7.5600 ± 2.5150 (33.3)	11.2785 ± 4.3841 (38.9)	27.8370 ± 31.9771 (114.9)	14.1726 ± 5.7297 (40.4)	45.1018 ± 57.4587 (127.4)
V_d_/F (L)	10.6184 ± 3.4254 (32.3)	14.2920 ± 4.8825 (34.2)	28.6062 ± 13.0076 (45.5)	28.7426 ± 10.2040 (35.5)	53.4886 ± 45.5859 (85.2)

AUC_0-t_, area under the concentration-time curve from time 0 to time t; AUC_0-∞_, area under the concentration-time curve from time 0 to infinity; C_max_, maximum serum concentration; CL/F, systemic clearance; CV%, coefficient of variation; PK, pharmacokinetic; SD, standard deviation; t_1/2_, terminal half-life; T_max_, time to peak concentration; V_d_/F, apparent volume of distribution.

*T_max_ was presented as median (min, max).

The slopes of AUC_0-∞_ and AUC_0-t_ were 0.9674 and 0.9669, respectively, in 75- to 300-mg doses of WBP216. The closeness of slopes to 1 indicates a linear trend to characterize PK characteristics. However, since the 90% CI of the slope was not completely within the confidence interval, the AUC_0-∞_ and AUC_0-t_ did not have a strict linear dynamic relationship with the dose. The slope of C_max_ was 0.6664 in the dose range of 75 to 300 mg, indicating no linear correlation between C_max_ and the dose.

### Immunogenicity

Positive anti-drug antibody (ADA) was observed in 1 subject in each WBP216 30-mg group (at baseline and week 20), WBP216 150-mg group (at week 12, 16, 20, and 24), and WBP216 300-mg group (at baseline). Anti-drug antibodies were detected in only one subject after dosing, indicating an acceptable immunogenicity profile. In all these 3 subjects, C_max_ and AUC were not reduced substantially compared to other subjects.

### Efficacy

**Figure 4:**
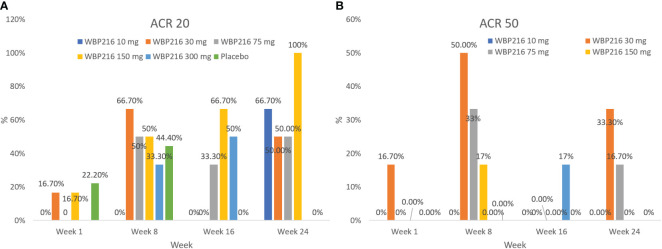
**(A)** ACR20 response rate in each cohort: efficacy analysis set; **(B)** ACR50 response rate in each cohort: efficacy analysis set ACR, American College of Rheumatology; DAS28, Disease Activity Score 28-joint assessment.

At week 1 (day 8), 11.1% (3.11%, 26.06%) of subjects achieved ACR20 and 2.8% (0.07%, 14.53%) of subjects achieved ACR50 response rates, while in the placebo group the ACR20 and ACR50 response rates were 22.2% (2.81%, 60.01%) and 0 (0.00, 33.63%), respectively. A gradual increase in ACR20 and ACR50 response rates was observed from week 4 (day 29) in the overall population but no substantial change in the placebo group. At week 24 (day 169), the ACR20 and ACR50 response rates for the overall study population were 38.9% (95% CI: 23.14, 56.54) and 8.3% (95% CI: 1.75, 22.47), respectively, while in the placebo group the rates were 0 (95% CI: 0.00, 33.63) and 0 (95% CI: 0.00, 33.63), respectively (**Figures 4A, B**).

With WBP216 treatment, the number of subjects with DAS28 scores ≤2.6 increased while the number of subjects with DAS28 scores ≥5.1 decreased, with no substantial change in the placebo group. At week 24 (day 169), 6 subjects (16.7%) had DAS28 scores ≤2.6, 4 subjects (11.1%) had DAS28 scores >2.6 and ≤ 3.2, 15 subjects (41.7%) had DAS28 scores > 3.2 and ≤ 5.1, and 2 subjects (5.6%) had DAS28 scores > 5.1 (**Table 4**).

**Table 4:** Summary of DAS28 disease activity: Efficacy analysis set.

Timepoint	Category	10 mg WBP (N = 3) n (%)	30 mg WBP (N = 6) n (%)	75 mg WBP (N = 6) n (%)	150 mg WBP (N = 6) n (%)	300 mg WBP (N = 6) n (%)	Placebo (N = 9) n (%)	Total (N = 36) n (%)
Baseline	>5.1	1 (33.3)	4 (66.7)	5 (83.3)	3 (50.0)	3 (50.0)	7 (77.8)	23 (63.9)
	>3.2 & ≤5.1	2 (66.7)	2 (33.3)	1 (16.7)	3 (50.0)	3 (50.0)	2 (22.2)	13 (36.1)
	>2.6 & ≤3.2	0	0	0	0	0	0	0
	≤2.6	0	0	0	0	0	0	0
Week 1	>5.1	1 (33.3)	1 (16.7)	4 (66.7)	1 (16.7)	3 (50.0)	7 (77.8)	17 (47.2)
	>3.2 & ≤5.1	2 (66.7)	4 (66.7)	2 (33.3)	3 (50.0)	3 (50.0)	2 (22.2)	16 (44.4)
	>2.6 & ≤3.2	0	0	0	0	0	0	0
	≤2.6	0	1 (16.7)	0	2 (33.3)	0	0	3 (8.3)
Week 4	>5.1	0	1 (16.7)	3 (50.0)	2 (33.3)	0	5 (55.6)	11 (30.6)
	>3.2 & ≤5.1	3 (100)	3 (50.0)	2 (33.3)	2 (33.3)	5 (83.3)	4 (44.4)	19 (52.8)
	>2.6 & ≤3.2	0	1 (16.7)	1 (16.7)	0	1 (16.7)	0	3 (8.3)
	≤2.6	0	1 (16.7)	0	2 (33.3)	0	0	3 (8.3)
Week 8	>5.1	0	1 (16.7)	0	1 (16.7)	0	2 (22.2)	4 (11.1)
	>3.2 & ≤5.1	3 (100)	2 (33.3)	3 (50.0)	1 (16.7)	3 (50.0)	7 (77.8)	19 (52.8)
	>2.6 & ≤3.2	0	1 (16.7)	1 (16.7)	3 (50.0)	0	0	5 (13.9)
	≤2.6	0	2 (33.3)	2 (33.3)	1 (16.7)	3 (50.0)	0	8 (22.2)
Week 12	>5.1	0	0	2 (33.3)	1 (16.7)	0	5 (55.6)	8 (22.2)
	>3.2 & ≤5.1	3 (100)	3 (50.0)	2 (33.3)	1 (16.7)	4 (66.7)	4 (44.4)	17 (47.2)
	>2.6 & ≤3.2	0	1 (16.7)	2 (33.3)	1 (16.7)	1 (16.7)	0	5 (13.9)
	≤2.6	0	2 (33.3)	0	2 (33.3)	1 (16.7)	0	5 (13.9)
Week 16	>5.1	0	0	2 (33.3)	0	0	0	2 (5.6)
	>3.2 & ≤5.1	0	0	3 (50.0)	3 (50.0)	3 (50.0)	0	9 (25.0)
	>2.6 & ≤3.2	0	0	1 (16.7)	0	1 (16.7)	0	2 (5.6)
	≤2.6	0	0	0	3 (50.0)	2 (33.3)	0	5 (13.9)
Week 20	>5.1	0	0	1 (16.7)	0	0	0	1 (2.8)
	>3.2 & ≤5.1	0	0	4 (66.7)	3 (50.0)	4 (66.7)	0	11 (30.6)
	>2.6 & ≤3.2	0	0	0	0	1 (16.7)	0	1 (2.8)
	≤2.6	0	0	1 (16.7)	3 (50.0)	1 (16.7)	0	5 (13.9)
Week 24	>5.1	0	0	1 (16.7)	0	1 (16.7)	0	2 (5.6)
	>3.2 & ≤5.1	2 (66.7)	4 (66.7)	4 (66.7)	2 (33.3)	3 (50.0)	0	15 (41.7)
	>2.6 & ≤3.2	1 (33.3)	1 (16.7)	0	1 (16.7)	1 (16.7)	0	4 (11.1)
	≤2.6	0	1 (16.7)	1 (16.7)	3 (50.0)	1 (16.7)	0	6 (16.7)

Baseline means last non-missing value before first dose.

Physician’s global assessment of disease activity scores and number of swollen and tender joints decreased from baseline in the WBP216 groups and slightly decreased from baseline in the placebo group. The subject’s global assessment of disease activity score also decreased from baseline in WBP216 groups, but increased in the placebo group.

## Discussion

In this phase Ia, randomized, placebo-controlled study, the analysis of safety data revealed an acceptable safety profile for WBP216 in 10- to 300-mg single dose. The most common AEs observed in this study included upper respiratory tract infection, increase in aspartate aminotransferase levels, alanine aminotransferase levels, blood alkaline phosphatase levels, blood creatine phosphokinase levels, blood thyroid stimulating hormone, and reticulocyte percentage, decrease in neutrophil count, hemoglobin, and white blood cell count, leukopenia, and dizziness, which is similar to other agents targeting IL-6 (24–26). Most of the AEs were of Grade 1 severity, and resolved without treatment. No TEAEs or TESAEs leading to withdrawal from the study, or leading to death during the study, were reported, indicating the safety and tolerability of WBP216 even at the highest dose of 300 mg.

With the increase in dose of WBP216, the AUC_0-∞_, AUC_0-t_, and C_max_ of serum WBP216 levels also increased. The slopes of AUC_0-∞_ and AUC_0-t_ were close to 1, indicating linear characteristics. However, perhaps due to the small sample size and inter individual variation, the 90% CI of the slope was not completely within the judgment interval, and it was not proved that there was a strict linear dynamic characteristic for AUC and C_max_. The t_1/2_ of treatment groups ranged from 980 to 1491 hours, which is significantly longer than the available anti-IL-6 inhibitor, tocilizumab (~29 hours) (27).

In a multicenter study, IL-6 levels increased after tocilizumab administration in patients with RA, reaching a maximum on day 4; levels then decreased slowly with repeated dosing (28). Also, in the present study, an increase in total IL-6 level was observed with a decrease in hs-CRP and ESR in all WBP216 groups from baseline with treatment. ADAs develop in up to one-third of the number of patients treated with biologic agents resulting in loss of efficacy in patients treated with biologics as shown in a study by Schaeverbeke et al. (29). In our study, only 1 subject in the WBP216 30 mg group had positive ADA results at baseline and at week 20, and 1 subject in the WBP216 300-mg group had positive ADA results at baseline and both were considered as false positive. Similar to our study, in a study by Sigaux et al. 91 samples from 40 patients with RA were analyzed for immunogenicity and none of the 91 samples showed persistent ADAs to tocilizumab and only 3 patients with RA showed transient and low titers of anti-tocilizumab ADAs (30).

ACR20 and ACR50 responses were observed in all groups, but no substantial change was observed in the placebo group. Due to the limited sample size in our study, it was difficult to interpret the efficacy of WBP216 based on the results of ACR response. A decrease in DAS28 scores was observed in all WPB216 dose groups, but there was no substantial change in the placebo group. The decrease was more remarkable in ≥30-mg groups than in 10 mg. This suggests that WBP216 had potential effect to decrease the disease activity in RA subjects, but it still needs further validation with more subjects to determine the most appropriate dosage.

This was the first clinical study to evaluate the safety, tolerability, PK, PD, and immunogenicity of WBP216 in the Chinese population. The acceptable safety profile and longer t_1/2_ of WBP216, which allows for a less frequent administration compared to other IL-6 inhibitors, could provide a new therapeutic option to the clinicians for the treatment of patients with RA. However, the study also has several limitations. This study was conducted only in Chinese patients, and hence, the results might not be generalizable to other geographic regions. Moreover, there was a lack of sample size calculation and the sample size was mainly based on the requirement of the CFDA for PK studies, and hence, the evaluation of WBP216 in a larger patient population over a longer treatment duration may provide further insights. Further studies with a larger population and longer follow-up period are required to substantiate these results.

## Conclusion

WBP216 demonstrated acceptable safety and immunogenicity profiles and longer t_1/2_, supporting a less frequent administration compared to other IL-6 inhibitors. Data obtained from this phase Ia study support further exploration and development of WBP216 in the treatment of patients with RA.

## Data availability statement

The original contributions presented in the study are included in the article/**Supplementary Material**. Further inquiries can be directed to the corresponding authors.

## Ethics statement

The studies involving human participants were reviewed and approved by the ethic committee of Peking Union Medical College Hospital, Chinese Academy of Medical Sciences and Beijing Hospital (site 1 approval number 2017BJYYEC-019-02/site 2 approval number: HS2017014/NMPA approval number: 2016L10654). The patients/participants provided their written informed consent to participate in this study.

## Author contributions

All authors contributed to the article and approved the submitted version.

## Supplementary material

Supplementary Table 1 Medical history, prior and concomitant medications.
