# Supplementary material for: Safety, tolerability, pharmacokinetics, pharmacodynamics, and efficacy of WBP216, a novel IL-6 monoclonal antibody, in patients with rheumatoid arthritis: A phase Ia randomized placebo-controlled study
Source: Front Immunol. 2023 Feb 28;13:1110992. doi: 10.3389/fimmu.2022.1110992 (PMC10011485; doi:10.3389/fimmu.2022.1110992)
Supplement: Supplementary Table 1 — Medical history, prior and concomitant medications. [file Table_1.docx]

**Supplementary Table 1. Medical History, Prior and Concomitant Medications**

| **Characteristics** | **N (%)** |
| --- | --- |
| Presence of ≥ 1 medical history | 32 (78.0%) |
| Metabolism and nutrition disorders | 11 (26.8%) |
| Eye disorders | 4 (9.8%) |
| Gastrointestinal disorders | 4 (9.8%) |
| Infections and infestations | 4 (9.8%) |
| Vascular disorders | 4 (9.8%) |
| Nervous system disorders | 3 (7.3%) |
| Ear and labyrinth disorders | 2 (4.9%) |
| Endocrine disorders | 2 (4.9%) |
| Hepatobiliary disorders | 2 (4.9%) |
| Investigations | 2 (4.9%) |
| Musculoskeletal and connective tissue disorders | 2 (4.9%) |
| Respiratory, thoracic and mediastinal disorders | 2 (4.9%) |
| Blood and lymphatic system disorders | 1 (2.4%) |
| Cardiac disorders | 1 (2.4%) |
| General disorders and administration site conditions | 1 (2.4%) |
| Immune system disorders | 1 (2.4%) |
| Renal and urinary disorders | 1 (2.4%) |
| Prior anti-rheumatics | 10 (24.4%) |
| Methotrexate | 7 (17.1%) |
| Others | 5 (12.2%) |
| Prednisone | 4, (9.8%) |
| Hydroxychloroquine | 3 (7.3%) |
| Leflunomide | 3 (7.3%) |
| Prior non-anti-rheumatics | 1 (2.4%) |
| Concomitant anti-rheumatics | 40 (97.6%) |
| Others | 36 (87.8%) |
| Methotrexate | 21 (51.2%) |
| Prednisone | 13 (31.7%) |
| Leflunomide | 12 (29.3%) |
| Hydroxychloroquine | 11 (26.8%) |
| Sulfasalazine | 1 (2.4%) |
| Concomitant non-anti-rheumatics | 20 (48.8%) |
| Unspecified herbal and traditional medicine | 5 (12.2%) |
| Macrolides | 3 (7.3%) |
| Non-pharmaceutical anti-rheumatics | 3 (7.3%) |
| Non-pharmaceutical non-anti-rheumatics | 7 (17.1%) |
